# A genome-wide analysis of nonribosomal peptide synthetase gene clusters and their peptides in a *Planktothrix rubescens *strain

**DOI:** 10.1186/1471-2164-10-396

**Published:** 2009-08-25

**Authors:** Trine B Rounge, Thomas Rohrlack, Alexander J Nederbragt, Tom Kristensen, Kjetill S Jakobsen

**Affiliations:** 1 University of Oslo, Department of Biology, Centre for Ecological and Evolutionary Synthesis (CEES), 0316 Oslo, Norway; 2 NIVA, Norwegian Institute for Water Research, 0411 Oslo, Norway; 3 University of Oslo, Department of Molecular Biosciences, 0316 Oslo, Norway; 4 University of Oslo, Department of Biology, Microbial Evolution Research Group (MERG), 0316 Oslo, Norway

## Abstract

**Background:**

Cyanobacteria often produce several different oligopeptides, with unknown biological functions, by nonribosomal peptide synthetases (NRPS). Although some cyanobacterial NRPS gene cluster types are well described, the entire NRPS genomic content within a single cyanobacterial strain has never been investigated. Here we have combined a genome-wide analysis using massive parallel pyrosequencing ("454") and mass spectrometry screening of oligopeptides produced in the strain *Planktothrix rubescens *NIVA CYA 98 in order to identify all putative gene clusters for oligopeptides.

**Results:**

Thirteen types of oligopeptides were uncovered by mass spectrometry (MS) analyses. Microcystin, cyanopeptolin and aeruginosin synthetases, highly similar to already characterized NRPS, were present in the genome. Two novel NRPS gene clusters were associated with production of anabaenopeptins and microginins, respectively. Sequence-depth of the genome and real-time PCR data revealed three copies of the microginin gene cluster. Since NRPS gene cluster candidates for microviridin and oscillatorin synthesis could not be found, putative (gene encoded) precursor peptide sequences to microviridin and oscillatorin were found in the genes *mdn*A and *osc*A, respectively. The genes flanking the microviridin and oscillatorin precursor genes encode putative modifying enzymes of the precursor oligopeptides. We therefore propose ribosomal pathways involving modifications and cyclisation for microviridin and oscillatorin. The microviridin, anabaenopeptin and cyanopeptolin gene clusters are situated in close proximity to each other, constituting an oligopeptide island.

**Conclusion:**

Altogether seven nonribosomal peptide synthetase (NRPS) gene clusters and two gene clusters putatively encoding ribosomal oligopeptide biosynthetic pathways were revealed. Our results demonstrate that whole genome shotgun sequencing combined with MS-directed determination of oligopeptides successfully can identify NRPS gene clusters and the corresponding oligopeptides. The analyses suggest independent evolution of all NRPS gene clusters as functional units. Our data indicate that the *Planktothrix *genome displays evolution of dual pathways (NRPS and ribosomal) for production of oligopeptides in order to maximize the diversity of oligopeptides with similar but functional discrete bioactivities.

## Background

Cyanobacteria produce a high number of chemically diverse oligopeptides exhibiting various types of bioactivities ranging from mild enzyme inhibition to initiation of acute toxic effects in pro- or eukaryotes [1]. More than 600 individual compounds have been described and probably many peptides have remained undiscovered. Most oligopeptides can be assigned to chemical classes, of which aeruginosins, anabaenopeptins, cyanopeptolins, microcystins, microginins, and microviridins are among the most recognized ones [2]. Some classes have well characterized biosynthetic pathways [3-6], while for others, hardly anything is known. The biological functions of cyanobacterial oligopeptides are unknown, despite knockout studies for microcystins and cyanopeptolins [3,4,7]. However, gene knockouts in the relevant cyanobacterial strains have turned out to be difficult. Thus, the link between a given oligopeptide and the gene cluster has generally been difficult to establish. Since the precise functions of the peptides are unknown, the reasons for the vast structural peptide diversity (both within- and between classes of oligopeptides) remain obscure.

Several of the oligopeptides classes are produced by nonribosomal peptide synthetases (NRPSs). NRPS pathways have been verified for microcystins [3,6,8], cyanopeptolins [4,9,10] and aeruginosins [5]. NRPSs have a modular structure with distinct activation domains (A-domains), thiolation domains (T-domains), and condensation domains (C domains) that are easily identified due to signature sequences [11]. Conserved sequence motifs also allow identification of additional modules and domains that often are present in NRPS enzyme complexes, including metyltransferases (M-domains), epimerases (E-domains), thioesterase (TE domains), halogenases, and ABC transporters. The oligopeptide products of NRPS gene clusters may be predicted *in silico *based on binding pocket analyses of A-domains [12,13], phylogeny and the co-linearity rule, i.e. the order of A-domains is co-linear with the amino acid sequence of the finished peptide [14]. Similar predictions of secondary metabolites from NRPS gene clusters have been performed successfully previously in *Streptomyces *([15]). Some oligopeptides are, however, synthesized ribosomally [16-18], and it is at present unknown if this is the case also for other classes. Due to insufficient genomic information this question has been hard to address.

Evolutionary and phylogenetic studies of individual NRPS gene clusters have revealed frequent horizontal gene transfer (HGT) between related strains [19-21]. This is likely to generate new or recurrent variants of the enzymatic modules, leading to a change in oligopeptide profiles. In addition to HGT (intergenomic recombination), recombination between sequences within the same genome (intragenomic) may occur even between different classes of NRPS clusters due to a general high genetic similarity (see Majewski and Cohan [22] and Papke *et al*. [23]) among the building blocks (i.e. the modules) [9,10,24] of NRPS gene clusters. Recombination events and point mutations may be reinforced by positive selection for the new variant, indicating that the new oligopeptide variant may have biological significance [25].

Welker *et al*, 2006 [2] indicated that production of oligopeptides is concentrated within certain genera of cyanobacteria. Why do most strains belonging to in the NRPS producing genera produce several classes of oligopeptides and multiple variants of the same oligopeptide class? Further, what is the biological significance of the many recombination events within the NRPS gene clusters? So far, most studies have investigated single NRPS operons (and their oligopeptides) or compared variants of the same class of operons in different strains. It is likely that all the different oligopeptides within a strain contribute to its survival and fitness, therefore the most relevant approach to take is to examine the entire genome of a strain for NRPS genes. Such an approach should establish relationship between each oligopeptide and operon, and consequently reduce the need for knock-out mutants. Furthermore, the possibility of ribosomal synthesis of some oligopeptide classes can be addressed. In addition, it will be possible to investigate whether exchange of modules between different classes of NRPS gene clusters within the same genome occurs.

Here, we have selected a *Planktothrix rubescens *strain, NIVA CYA 98, that produces all major classes of oligopeptides according to Welker *et al *[2] and shotgun sequenced the genome by massive parallel pyrosequencing (454) to high depth (18.5×). We have characterized the oligopeptides of this strain in detail by LC-MS-MS. This has enabled a correlation of NRPS gene clusters found within the genome with the identified oligopetides. A putative gene cluster could be identified for all structurally characterized oligopeptides. However, for two NRPS gene clusters no oligopeptide were detected. Overall, the data show that the combined LC-MS-MS and 454 genome sequencing is a very powerful approach for finding putative secondary metabolite genes.

## Results

### Oligopeptides produced by *Planktothrix *NIVA CYA 98

Prior to 454-genome sequencing we performed a LC-MS-MS mass spectroscopic fragmentation analysis of *Planktothrix *NIVA CYA 98. This analysis revealed thirteen oligopeptides representing all major oligopeptide classes (Additional file 1: Table S1, Additional file 2: Figure S1). Aeruginosins, anabaenopeptins, microcystins, and microginins were found to occur with at least two variants each, typically differing by one amino acid or, as for microginins, by the presence/absence of Cl in the molecule (Additional file 1: Table S1). An aeruginosin with a previously unreported molecular mass of 593.5 Da [M+H]^+ ^was detected, but not fully elucidated. The oligopeptide nature of a second unknown compound with a mass of 1971.8 Da [M+H]^+ ^could not be verified due to the production of immonium ions in MS fragmentation experiments. The molecular mass suggested that the oligopeptide might be a microviridin, which is the only known cyanobacterial oligopeptide class within this size range. A full structural elucidation was not attempted.

### The genome of *Planktothrix *NIVA CYA 98 and detection of NRPS gene clusters

**Figure 1:**
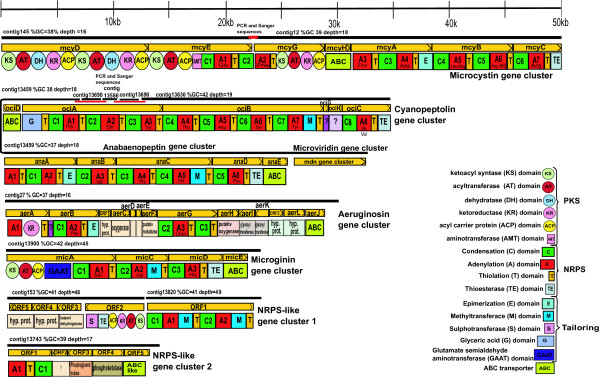
**Organization of the NRPS gene clusters found in the *Planktothrix *NIVA CYA 98 genome**. Gene clusters shown are: microcystin [EMBL: AM990462], cyanopeptolin, anabaenopeptin and microviridin (putative ribosomal produced oligopeptide) on one contig [EMBL: AM990463], aeruginosin [EMBL: AM990465], microginin [EMBL: AM990464] synthetase gene clusters and two gene clusters with unassigned oligopeptides [EMBL: AM990466 and AM990467]. Contigs from the assembly of 454 reads are illustrated with black lines and gaps closed with Sanger sequencing are illustrated with red lines. Gene names, transcription directions and approximate sizes are illustrated with yellow boxes with arrows. Adenylation- (red; showing *in silico *predicted activated amino acids), condensation- (green), thiolation-(yellow), methyltransferase- (blue), epimerase- (turquoise) glyceric acid- (lilac), halogenation- (purple) and termination domains (grey) are shown by their first letter abbreviations. Polyketide domains are shown as circles. The ABC transporter genes (light green) are connected to six of the seven gene clusters.

The 454 pyrosequencing-generated genomic sequence dataset consisted of 570912 reads, with the average read length at 262 bp, totaling 150 Mb (Short read archive SRA008127). An assembly produced 692 contigs larger than 500 bp. The average contig size was 8240 bp and the largest contig was 129206 bp. The genome size was estimated at 5.5 Mb with an average GC content of 40.0%. An average sequencing depth of 18.5× was obtained, and the gaps between contigs were no larger than 100 bp within the NRPS gene clusters regions (positions shown in Figure 1). In this work, we were able to detect all NRPS gene clusters by performing BLAST [26] comparisons of the entire genome assembly with a relevant selection of NRPS domains. We have focused our assembly on the putative NRPS sequences. For these sequences we have performed closing of gaps (5 gaps, maximum gap length of 7 bp) and confirmation of correct assembly with PCR and Sanger sequencing (total 5700 bp).

Gene clusters for all structurally determined oligopeptides detected by LC-MS/MS were found among the contigs. These were identified utilizing BLAST searches with characterized A- and C-domains as query sequences. A BLASTn search identified 30 distinct regions with high similarity to previously characterized A-domains (E value < 10^-10^). Other regions were similar to known A-domains (E value range 10^-10 ^to 10^-1^), but lacked typical A-domain characteristics. Twenty-eight regions similar to known NRPS C-domains (E value < 10^-10^) were also identified using BLASTn search. In addition, a number of other domains and genes connected to NRPSs were found, including those encoding ABC-transporters.

### Seven distinct NRPS gene clusters present in *Planktothrix *NIVA CYA 98

The annotation of the genome uncovered seven distinct NRPS clusters (Figure 1 and Additional file 3: Table S1) distributed on twelve contigs. Three NRPS gene clusters – *aer*, *oci*, and *mcy *– were highly similar in domain structure and sequence to clusters previously associated with the production of aeruginosins (96% identity to [GenBank: AM071396]) [5], cyanopeptolins (95% identity to [GenBank: DQ837301]) [4,9,10], and microcystins (99% identity to [GenBank: AJ441056]) [3,6,8], respectively. The oligopeptide structures predicted by gene identity and order were in agreement with the structural data obtained in MS fragmentation experiments (Additional file 1: Table S1). We are therefore confident that the NRPS encoded by clusters *mcy*, *oci*, and *aer *are responsible for the synthesis of two demethylated microcystins (*mcy*), the cyanopeptolin oscillapeptin G (*oci*), and two aeruginosins (*aer*) by NIVA CYA 98.

A large NRPS gene cluster was situated in close proximity to the cyanopeptolin gene cluster (*oci*), but transcribed in the opposite direction on the same contig (contig 13459). The novel gene cluster (*ana*ABCDE) encodes six basic NRPS modules, including an epimerase and a methyltransferase domain, and an ABC transporter (Figure 1). The size, organization and amino acid specificity of A-domains predicted a link between this gene cluster and production of anabaenopeptins. A reliable prediction of amino acid specificity in the AnaA-A1 and AnaA-A2 domains was not possible. However, the occurrence of lysine in all anabaenopeptins suggested that the protein complex named AnaABCDE was responsible for production of all four anabaenopeptin variants – with AnaA-A2 domain activating Lys and relaxed amino acid specificity of the AnaA-A1 (Tyr/Arg) and AnaB-A3 (Val/Ile).

The *Planktothrix *CYA 98 genome also contains another new NRPS polyketide hybrid gene cluster (*mic*ACDE). The gene cluster encodes three basic NRPS modules, a polyketide module and an aminotransferase. *In silico *analyses of the novel MicACDE suggested that the gene cluster corresponded to the microginin oscillaginin B produced by NIVA CYA 98. Oscillaginin A is chlorinated and thus a halogenase is predicted to be part of the biosynthetic pathway. However, BLAST searches could not identify a complete halogenase in the gene cluster or in the entire genome.

The products of the two remaining NRPS gene clusters could not be assigned to oligopeptides detected by LC-MS-MS. NRPS-like gene cluster 1 did not contain an ABC transporter and the assembly of contig 153 (polyketide) and 13820 (peptide synthetase) into a single contig displayed low sequence depth in the contig breakpoint. Non-NRPS genes flanked NRPS-like gene cluster 2. The comparison of oligopeptides detected by mass spectroscopy and predicted by genetic analysis did not identify any NRPS gene clusters associated to the production of oscillatorin and the putative microviridin.

### Multi copy NRPS gene clusters

To assess the quality of the contigs containing NRPS gene clusters, sequence depth per base were analyzed (Additional file 1: Table S1). Contig 13900 (length 32 kb) containing the 21 kb microginin gene cluster and the contig containing NRPS-like gene cluster 1 displayed higher sequence depth than the average sequence depth of the genome. Higher sequence depth per base is expected to be observed when reads from repeats and duplicated regions are collapsed into a single contig during the assembly process. The sequence depth per base of the microginin contig (13900) indicated three copies and the read-alignment also showed about 28 sites to be variable among the reads (Additional file 3: Figure S1), were each read variant was represented by approximately 1/3 of the reads covering that site. The 3X copy number of the microginin gene cluster was confirmed by real-time PCR in an independent study (see Nederbragt AJ, Rounge TB, Jakobsen KS: Identification and quantification of genomic repeats and sample contamination in assemblies of 454 pyrosequencing reads, submitted).

PCR and Sanger sequencing showed that contig 13690 was present in two repeats within the cyanopeptolin gene cluster. This contig (13690) also showed two-fold higher sequence depth per base (Additional file 1: Table S1) and which is in agreement with the sequence depth per base/number of gene copy relationship as shown by Nederbragt AJ, Rounge TB, Jakobsen KS: Identification and quantification of genomic repeats and sample contamination in assemblies of 454 pyrosequencing reads, submitted.

Altogether, the NRPS gene clusters accounted for 4.1% (226 kb/5500 kb) of the entire *Planktothrix *genome when considering the putative duplication of contigs 153, 13820 and 13900. The typical GC content of NRPS clusters ranged from 37 to 42% (Additional file 1: Table S1).

### Ribosomal synthesis of microviridin and oscillatorin

**Figure 2:**
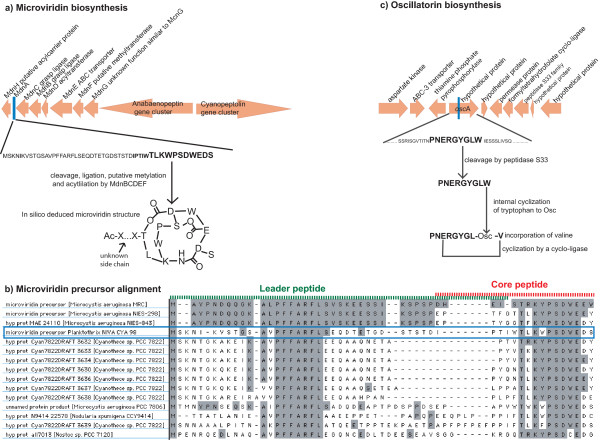
**Proposed microviridin and oscillatorin biosynthetic pathways**. Orange arrows, with protein name and predicted function above, illustrate the genes size and transcription direction. a) MdnA is proposed as microviridin precursor. MdnCB are predicted to ligate the ring structures, MdnD is responsible for acetylation. A microviridin with unknown side chain was predicted based on the precursor sequence (MdnA) and gene similarities to the *Microcystis *microviridin gene clusters [16]. b) The alignment of sequences with high similarities to *Microcystis *MdnA shows two highly conserved regions in the leader peptide (green dashed line) and core peptide (red dashed line). The leader-core transition is unknown. c) oscA is proposed as the oscillatorin precursor encoding 619 amino acids, includes eight of the 10 amino acids in oscillatorin (bold text). The tryptophan is suggested as a precursor to oscillatoric acid (Osc). Based on in silico analyses of the gene cluster, the oscillatorin precursor is likely to be cleaved by peptidase S33. Modification of tryptophan and addition of valine is carried out by unknown enzymes. A putative cyclo-ligase is may be involved in cyclization.

Since NRPS gene cluster candidates for microviridin and oscillatorin synthesis could not be found, putative ribosomal synthesized precursor genes were searched for and found in the genes *mdn*A and *osc*A, respectively. BLAST identified a gene cluster *mdn*ACBDEF (Figure 2a and Additional file 3: Table S1), in close proximity to the anabaenopeptin and cyanopeptolin gene clusters, with high similarities to microviridin biosynthesis genes from *Microcystis *[17]. A putative microviridin precursor MdnA included a leader and core peptide region with conserved motives (Figure 2b). Based on *in silico *analyses of the MdnA core peptide and comparison to the microviridin biosynthetic pathway in *Microcystis*, a microviridin biosynthesis pathway in *Planktothrix *NIVA CYA 98 can be suggested. The pathway includes cleavage, ligation, acetylation and possible metylation and results in an *in silico *predicted microviridin with an unknown side chain. The genomic island, constituting the microviridin gene cluster and the two NRPS gene clusters *ana *and *oci*, we term an oligopeptide island. Interestingly, it includes both putative ribosomal and NRPS biosynthetic pathways.

tBLASTn search identified an 1853 bp open reading frame in the *Planktothrix *genome that encoded an eight amino acid sequence identical to Pro-Asn-Glu-Arg-Gly-Tyr-Gly-Leu sequence of oscillatorin. The protein, here named OscA (accession AM990468), has unknown function. The ninth amino acid flanking Leu is tryptophan suggested by Sano and Kaya [27] to be modified to oscillatoric acid (Osc), another part of oscillatorin. The probability of a coincidental occurrence of the nine amino acid sequence is 1.0 × 10^-12 ^(see calculation in Methods) and we therefore suggest that the main part of oscillatorin is produced ribosomally as an integral part of this protein. Similar schemes have been shown for other oligopeptides [16-18]. Thus, we propose a biosynthetic pathway for oscillatorin involving ribosomally encoded amino acids (Pro-Asn-Glu-Arg-Gly-Tyr-Gly-Leu), plus a valine of unknown origin, and cyclization of the oligopeptide by a modifying enzyme possibly encoded by the putative formyltetrahydropholate cycloligase found in the *osc *gene cluster (Figure 2c). A BLAST search showed similarity to a hypothetical protein L8106_04601 from Lyngbya sp. PCC 8106 (53% identity). Interproscan [28] identified motifs similar to pectate lyases (Pfam CL0268).

### Intra-genomic evolutionary processes unveiled by phylogeny

**Figure 3:**
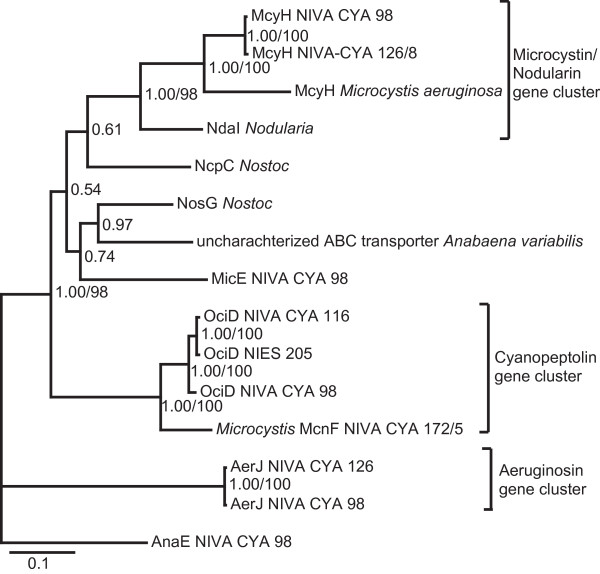
**ABC transporter tree constructed using Bayesian inference**. Support values for the nodes are Bayesian posterior probability and NJ bootstrap replicates above 50%. Genus origin is denoted for taxa other than Planktothrix. Accession numbers for the sequences are listed in Additional file 3: Table S3. The CpRev substitution model and gamma-shaped distribution were used in the Bayesian analyses. The MCMC chains were carried out for 4 million generations with sampling of trees every 100 generations, removing the first 3000 trees.

Phylogenetic analyses of A-, C-, M-, E-domains (Additional file 3: Figures S2, S3, S4 and S5) and NRPS-ABC-transporter genes (Figure 3) including the *Planktothrix *CYA 98 NRPS sequences, supplemented with other NRPS sequences, showed clustering according to gene cluster type rather than strain or genera. A- and C- domains also showed functional clustering according to amino acid specificity and position in the gene cluster, respectively (Additional file 3: Figures S2 and S3). The phylogenetic analyses including only *Planktothrix *CYA98 domains indicated the same clustering according to gene cluster and position. However, due to the low number of sequences and few variable sites, the topology did not receive sufficient support (data not shown). Split decomposition analyses were used to identify conflicting phylogenies, which represent recombination events (Additional file 4: Figure S1). Splits analyses did not indicate any conflicting phylogenies between domains of the NRPS gene clusters that were determined to be statistic significant by the Phi test analyses (Additional file 4: Figure S1), indicating no recombination events.

## Discussion

### Metabolomics and the genome-wide approach

Here, we show that combining information about all oligopeptides (from LC-MS-MS) with whole genome sequencing of a *Planktothrix *strain is a powerful method to deduce the relations between oligopeptides and the corresponding NRPS gene clusters Identification of putative ribosomal pathways of oscillatorin and microviridin further demonstrates the power of the genome-wide approach. All sequence information needed to identify and characterize the oligopeptide gene clusters was derived from *de novo *shotgun sequencing of a previously unsequenced cyanobacterial genus, *Planktothrix*. The gene clusters were assembled to completion by Sanger-sequencing only a few short PCR fragments. The *Planktothrix *strain was not axenic, but *in silico *analyses of the 454-sequences solved this potential problem (Nederbragt AJ, Rounge TB, Jakobsen KS: Identification and quantification of genomic repeats and sample contamination in assemblies of 454 pyrosequencing reads, submitted). The gene clusters were identified without making an effort to assemble the complete genome. This is the most complete oligopeptide investigation in a single cyanobacterial strain to date, and we demonstrate here that the genome-wide approach using high-throughput sequencing combined with structural determination of the metabolites has the potential to accelerate oligopeptide research and increase our understanding of secondary metabolite synthesis in general.

### Link between NRPS gene clusters and oligopeptides

*In silico *analyses of the microcystin, cyanopeptolin and aeruginosin gene clusters correlate with the produced microcystin (desmethyl-RR), oscillapeptin G (cyanopeptolin) and aeruginosin A and a new aeruginosin variant detected by the MS analyses.

The link between the novel *ana *gene cluster, situated in close proximity to the cyanopeptolin gene cluster, and the anabaenopeptins is highly likely due to the correlation between the bioinformatic predictions and the LC-MS-MS deduction of the anabaenopeptins. It is likely that AnaABCDE produce all four anabaenopeptin variants due to relaxed specificity of the AnaA1 and AnaA2 domain. The epimerase domain suggests a D-amino acid in the A2 position, corresponding to the conserved Lysine in the oligopeptide, in agreement with structural analyses of anabaenopeptins [29]. However, the mass spectrometry analysis cannot differentiate between the D-and L-configurations of amino acids.

*In silico *analyses of MicACDE show that this gene cluster corresponds to the oscillaginin B produced by the strain. The chlorinated oscillaginin A requires an additional halogenase, but we were unable to detect a complete oligopeptide halogenase in the genome. The chlorine atom is attached to the polyketide part of the molecule, so a potential different halogenase outside the contig or in *trans *may be involved.

The microcystin, cyanopeptolin, and aeruginosin and the newly discovered anabaenopeptin and microginin gene clusters all share many features including ABC transporter genes. The function of NRPS ABC-transporters has not been determined, but it has been suggested that they are involved in the export of oligopeptides from the cells. Additionaly, the ABC transporter *mcy*J is shown to be essential for microcystin production [30]. All five NRPS gene clusters are co-linear and consist of very similar NRPS and PKS modules. Compared to previously characterized gene clusters, we observed small changes in aeruginosin gene cluster architecture [5], new tailoring genes/domains (*oci*G and *oci*H encoding unknown functions) and an additional NRPS module in the cyanopeptolin gene clusters (eight NRPS modules cyanopeptolin gene cluster, in contrast to previously described seven modules [10]) and a *mcy*A gene without a methyltransferase in the *mcy *gene cluster [31]. NRPS-like gene cluster 1, lacking an ABC transporter and therefore believed to be incomplete and NRPS-like gene cluster 2, containing only one NRPS encoding module, could not be linked to oligopeptides.

An almost three-fold higher sequencing depth per base and abundance in real-time PCR analysis suggested three copies of the microginin and the NRPS-like 1 gene clusters (Nederbragt AJ, Rounge TB, Jakobsen KS: Identification and quantification of genomic repeats and sample contamination in assemblies of 454 pyrosequencing reads, submitted). The variations (point mutations) in the reads detected in the microginin gene clusters point towards some sequence variation between the copies.

### Independent NRPS gene cluster evolution and finding of an oligopeptide island support similar, but discrete, oligopeptide functions

The phylogenetic analyses showed individual functional domains within a NRPS class to be more closely related, compared to NRPS modules from other NRPS classes within the same strain. This indicates independent evolution of each gene cluster, in line with data from single NRPS studies [9,10,25]. Our results therefore indicate that NRPS domain evolution is influenced more by functional constraints acting on the domains, than by the evolutionary relationships between strains. ABC transporters genes were shown to evolve together with their respective gene clusters as sign of a functional unit. Consequently, they are useful for studying NRPS gene cluster evolution. While the general module architecture was conserved within a gene cluster type, frequent tailoring domain rearrangements were shown resulting in new features like the G-domain, and OciH of the cyanopeptolin and partial halogenases in OciG and AerB. A module rearrangement by intra-gene cluster recombination (identical *oci*A C2 and C3) seems likely in the cyanopeptolin gene cluster.

An oligopeptide island – a parallel to pathogenicity islands including the three closely located oligopeptide gene clusters *oci, ana *and *mdn *(4597 and 2139 bp distance between gene clusters respectively) was observed. Co-localization of cyanobacterial oligopeptide gene clusters has not been reported previously and may indicate a common target or class of targets for the peptides. There were no further signs of closely linked NRPS gene clusters. However, a completely assembled genome is needed to determine the relative positions of the NRPS gene clusters to each other.

### Dual origin of oligopetides through NRPS and ribosomal pathways

If oscillatorin and microviridin were produced by NRPSs, the corresponding gene clusters would be large and thus easily detected in the genome by this method. These oligopeptides contain only L-amino acids suggesting a possible ribosomal pathway with subsequent modification and cyclization. It is therefore not unexpected that we have identified genes that a likely to ribosomally encode the precursor of oscillatorin and microviridin. The high similarity between the microviridin (*mdn*) gene cluster presented here and in two *Microcystis *strains [17] strongly suggest that the *mdn *gene cluster produce microviridin. However the length of the side chain, modifications of amino acids and occurrence of additional functional groups are unknown. Ribosomal pathways through the activity of a set of processing enzymes have been shown for microviridins [17] microcyclamide [18] and patellamides [16], demonstrating that at least two fundamentally different biosynthetic pathways are used by cyanobacteria to produce oligopeptides.

## Conclusion

The present study suggests that NRPS gene clusters encoding the synthesis of aeruginosins, anabaenopeptins, cyanopeptolins, and microginins evolve independently of one another. Using phylogenetic methods we showed that there are low levels of recombination between similar modules in functionally different NRPS gene clusters within one and the same strain. The recombination levels have not been possible to assess without this genome-wide sequencing approach. In addition, the strain NIVA CYA 98 features a likely ribosomal production of oscillatorin and microviridin. All of these six oligopeptide classes are known as potent inhibitors of different types of proteolytic enzymes, including serine proteases, metallo-proteases, and carboxypeptidases [27,32-37]. This strongly suggests dual pathway of evolution of protease inhibitors in *Planktothrix *NIVA CYA 98, allowing different rates of evolution for each NRPS and ribosomal oligopeptide gene clusters. Elucidation of evolutionary rates between the different oligopeptide gene clusters, in particular NRPS- and ribosomal derived, requires further studies. It is tempting, however, to attribute the dual evolution and high diversity of protease inhibitors in NIVA CYA 98 to an "arms-race" between unknown pathogens, competitors or grazers in cyanobacteria [38,39].

## Methods

### Cyanobacterial culture and growth conditions

*Planktothrix rubescens *NIVA CYA 98 was isolated in 1982 from Lake Steinsfjorden, Norway. The strain has been maintained in continuous culture in the NIVA culture collection of Algae in Z8 medium and light at a photon flux density of 10 μmol m^-2^s^-1^, and a light-dark cycle of 12:12 hours.

### Oligopeptide analysis

Oligopeptides were extracted from filters with cultured *Planktothrix *after lyophilisation using 50% MeOH as described previously [37]. For the detection and identification of oligopeptides, Liquid Chromatography Mass Spectroscopy (LC-MS-MS) was used. The MS-MS detector was run in total scanning mode for the mass range of 500 to 2000 Da during the entire Waters Acquity Ultra-performance Liquid Chromatography (UPLC) linear gradient (from 10% to 45% acetonitrile in water, both containing 0.1% formic acid, within 10 minutes at a flow rate of 0.25 mL min^-1^). Compounds with a molecular mass within the range of 500–2000 Da were further analyzed in fragmentation experiments with the detector in daughter ion scanning mode.

The structural elucidation of cyanobacterial oligopeptides on the basis of MS fragmentation experiments were identical to previously described studies [9,10,40,41].

### Sequencing and bioinformatics

DNA was extracted from cells frozen in isopropanol using a phenol-chloroform extraction. Massive parallel pyrosequencing was performed according to manufacturer's protocol on two PicoTiter Plates with a GS FLX instrument performed at Roche Penzburg, and at University of Oslo, Norway. PCR and Sanger-sequencing with specific primers (Additional file 3: Table S2) was used to close two gaps in the NRPS gene clusters and confirm the correct assembly of the cyanopeptolin gene cluster which was not possible to assemble with only 454 sequences. The gsAssembler 1.1.02 program (Roche-454, Basel, Switzerland) was used to assemble the 454-reads in contigs at default settings.

Axenic *Planktothrix *cultures are very difficult to obtain. A BLASTn search against the 16S rDNA database [42,43] confirms the contamination in *Planktothrix *NIVA CYA 98. 16S sequences similar to *Planktothrix, Pseudodevosia insulae*, *Mesorhizobium*, *Flavobacterium psychrophilum *and uncultured cyanobacteria were identified. However, due to large domination of *Planktothrix *in the culture only small fragments of other genomes were sequenced, and these sequences were not assembled in to large contigs due low number of reads. BLASTn of contigs showing atypical GC content and/or low sequence depth per base were shown to be contamination (Nederbragt AJ, Rounge TB, Jakobsen KS: Identification and quantification of genomic repeats and sample contamination in assemblies of 454 pyrosequencing reads, submitted).

Identification of contigs containing NRPS domains was performed with BLAST searches at the UiO Bioportal  with cyanopeptolin and microcystin A- and C-domains as query sequences, and confirmed using blast2go against the non-redundant protein sequences (nr) database. ORFfinder [44] and Vector NTI (Invitrogen, Carlsbad, USA) were used to find and translate open reading frames (ORF). Identification of domains, binding pocket signatures and A-domain substrate specificities were performed using the NRPS database [45]. A-domain specificities were also substantiated using phylogenetic analysis (Additional file 3: Figure S2). Functional predictions of other genes in connection with NRPS gene clusters were determined using BLAST, and conserved motifs searches using InterProScan [28]. A-, C-, M-, E- domains and ABC transporter gene Bayesian inference and neighbor joining (NJ) phylogeny was performed as described in Rounge *et al *[10] using MEGA 3.1 [46], MrBayes 3.1 [47], ProTest [48] and the UiO Bioportal. In addition to all the NRPS-domains from *Planktothrix *NIVA CYA 98, the phylogenetic analyses also include NRPS domains from other cyanobacteria (M-domain phylogenies also include other bacteria)(Accession numbers for the sequences used in the phylogenetic analyses are listed in Additional file 3: Table S3). The additional domains from other strains showed that the NIVA CYA 98 domains cluster in several different groups. Split decomposition analyses were performed using SplitsTree4 [49] with default settings (uncorrectedP method) and 1000 bootstrap replicas, and Phi test for recombination [50]. The probability of a coincidental encoded amino acid (P_aa_) is the number of codons encoding the amino acid divided by the total number of possible codons (i.e. 64). The probability of a coincidental occurrence of an amino acid sequence is P = P_aa _1 × P_aa _2×...P_aa _n. The coincidental occurrence of the amino acid sequence of oscillatorin is therefore P = (4/64)×(2/64)×(2/64)×(6/64)×(4/64)×(2/64)×(4/64)×(6/64)×(1/64) = 1.0 × 10^-12^. This calculation does not take into account codon usage bias.

%GC content and sequencing depth of each base, were determined from the alignment file utilizing a custom perl scripts.

## Authors' contributions

TBR carried out the genetic experimentations and TR carried out all MS experiments and oligopeptide analyses. All authors have contributed to the experimental and analytical design. TBR and AJN performed the sequencing, bioinformatics and phylogenetic analysis TBR, KSJ, AJN, TK and TR wrote the manuscript. All authors have read and approved the final manuscript.

## Supplementary Material

Additional file 1 **Overview of oligopeptides and gene clusters**. Table S1: Overview of oligopeptides and gene clusters. Click here for file

Additional file 2 **Oligopeptide structures**. Figure S1: Oligopeptide structures of Oscillaginins, Aeruginosins, Anabaenopeptin A, Microcystins-RR, Oscillapeptin G and Oscillatorin, The structure of the putative microviridin is not given since no structural elucidation has been conducted. Click here for file

Additional file 3 **NRPS protein sequence data, primers and accession numbers**. Table S1: Proteins encoded by the NRPS gene clusters. Table S2: PCR and sequencing primers. Table S3: Accession numbers for the sequences used in the phylogenetic analyses. Figure S1: Variation in the microginin gene cluster. Figure S2: A-domain tree – confirms amino acid activation. Figure S3: Bayesian C-domain tree. Figure S4: E-domains phylogeny. Figure S5: M-domains phylogeny. Click here for file

Additional file 4 **Splits decomposition analyses**. Figure S1: a) Splits decomposition analysis of A-domains from *Planktothrix *CYA 98. b) Splits decomposition analysis of C-domains from *Planktothrix *CYA 98. c) Split decomposition analysis of M-domains from *Planktothrix *CYA 98. d) Splits decomposition analysis of E-domains. e) Splits decomposition analysis of ABC transporters. Click here for file
