# Supplementary material for: A genome-wide analysis of nonribosomal peptide synthetase gene clusters and their peptides in a Planktothrix rubescens strain
Source: BMC Genomics. 2009 Aug 25;10:396. doi: 10.1186/1471-2164-10-396 (PMC2739229; doi:10.1186/1471-2164-10-396)
Supplement: Additional file 1 — Overview of oligopeptides and gene clusters. Table S1: Overview of oligopeptides and gene clusters. [file 1471-2164-10-396-S1.pdf]

Additional file 1: Overview of oligopeptides and gene clusters.

Oligopeptides				Gene clusters			
Oligopeptide	Class	Molecular Mass $[M+H]^+$(Da)	Amino acid sequence	*In silico* predicted amino acid sequence	Encoded by gene clusters/ on contigs	GC%	Average sequence depth per base
Oscillaginin B	microginins	581.4	Ahda*-Ser-MeVal-HTyr	Polyketide-Ser-Val-Hty	*mic*ACDE/13900	42	45
Oscillaginin A	microginins	615.5	Cl-Ahda-Ser-MeVal-HTyr	Polyketide-Ser-Val-Hty	*mic*ACDE/13900	42	45
New aeruginosin	aeruginosins	593.5	Pla**-Phe-Choi***-?	Pla-Phe-Choi-Aeap?	*aer*ABCDGHIJ/27	37	16
Aeruginosin A	aeruginosins	617.5	Pla**-Phe-Choi***-Aeap#	Pla-Phe-Choi-Aeap?	*aer*ABCDGHIJ/27	37	16
Anabaenopeptin B	anabaenopeptins	837.5	cyclo(Phe-MeAla-Htyr-Val-Lys)-Arg	Phe-MeAla-Hty-Val-?-?	*ana*ABCDE/13459	38	18
Anabaenopeptin A	anabaenopeptins	844.5	cyclo(Phe-MeAla-Htyr-Val-Lys)-Tyr	Phe-MeAla-Hty-Val-?-?	*ana*ABCDE/13459	38	18
Anabaenopeptin F	anabaenopeptins	851.5	cyclo(Phe-MeAla-Htyr-Ile-Lys)-Arg	Phe-MeAla-Hty-Val-?-?	*ana*ABCDE/13459/	38	18
Oscillamid Y	anabaenopeptins	858.5	cyclo(Phe-MeAla-Htyr-Ile-Lys)-Tyr	Phe-MeAla-Hty-Val-?-?	*ana*ABCDE/13459/	38	18
[Dha^7]Mcyst-LR	microcystins	981.5	cyclo(Ala-Leu-MeAsp-Arg-Adda##-Glu-Dha)	Ala-Leu/Arg-MeAsp-Arg-Adda-Glu-Dha	*mcy*DEGHABC/ 145 and 12	38 and 39	16 and 18
[Dha^7]Mcyst-RR	microcystins	1024.5	cyclo(Ala-Arg-MeAsp-Arg-Adda##-Glu-Dha)	Ala-Leu/Arg-MeAsp-Arg-Adda-Glu-Dha	*mcy*DEGHABC/ 145 and 12	38 and 39	16 and 18
Oscillapeptin G	cyanopeptolins	1112.6	cyclo(Leu-Ahp###-Thr-MeTyr-Ile-Thr)-Gln-Htyr-GA	Hty-Ahp-Thr-MeTyr-Ile/Val-Thr)-Gln-Htyr-GA	*oci*ABHC/13459, 13690 and 13586	38, 40 and 39	18, 34 and 19
Oscillatorin	not specified	1240.5	cyclo(Pro-Asn-Glu-Arg-Gly-Tyr-Gly-Leu-Osc¤-Val)	Phe-Asn-Glu-Arg-Gly-Tyr-Gly-Leu-Osc¤-	*osc*A/109	39	17
Putative microviridin	microviridins	1971.8	unknown	Ac-X…X-Thr-Leu-Lys-Trp-Pro-Ser-Asp-Trp-Glu-Asp-Ser	*mdn*ACBDEF/13459	38	18
				Sulphate-polyketide-Me amino acid-Me amino acid	NRPS-like gene cluster 1 /153 and 13820	41 and 41	48 and 49
				Amino acid (modified?)	NRPS-like gene cluster 2 /13743	39	17

Table S1: Oligopeptides produced by *Planktothrix* NIVA CYA 98, oligopeptide class, molecular mass for oligopeptide and amino acid sequence of oligopeptide are listed on the left side. Gene cluster *in silico* predicted oligopeptide sequence, names, contig number %GC and average sequence depth pr base of each gene cluster are listed on the right side. *Ahda: 3-amino-2-hydroxy-decanoic acid; **Pla: phenyl-lactic acid; ***Choi: 2-carboxy-6-hydroxyoctahydroindole; # Aeap: 1-amidino-2-ethoxy-3-aminopiperidine; ##Adda: (2S, 3S, 8S ,9S)-3-amino-9-methoxy-2,6,8-trimethyl-10-phenyldeca-4,6-dienoic acid; ###Ahp: 3-amino-6-hydroxy-2-piperidone; ¤Osc:(3a-cis)-1,2,3,3a,8,8a-Hexahydro-3a-(3-methyl-2-butenyl)-pyrrolo[2,3-b]indol-2-carboxylacid). X…X: unknown sidechain. Zigzag lines illustrate the putative relationship between gene – oligopeptide with regard to microviridin and oscillatorin.
