# Supplementary material for: A genome-wide analysis of nonribosomal peptide synthetase gene clusters and their peptides in a Planktothrix rubescens strain
Source: BMC Genomics. 2009 Aug 25;10:396. doi: 10.1186/1471-2164-10-396 (PMC2739229; doi:10.1186/1471-2164-10-396)

Additional file 2: Oligopeptide structures

Figure S1: Oligopeptide structures of Oscillaginins, Aeruginosins, Anabaenopeptin A, Microcystin-RR, Oscillapeptin G and Oscillatorin. The structure of the putative microviridin is not given since no structural elucidation has been conducted.

Oscillaginins

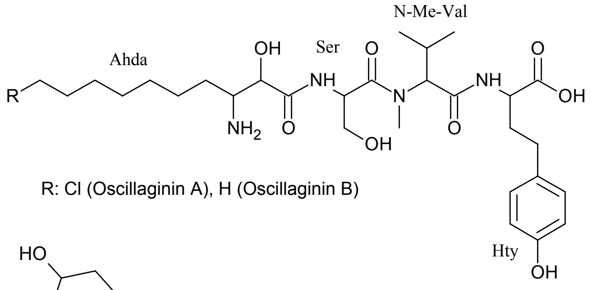

R: Cl (Oscillaginin A), H (Oscillaginin B)

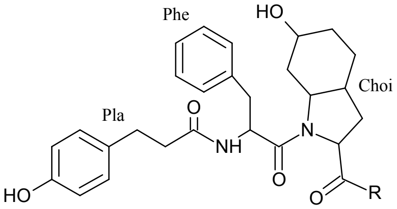

Aeruginosins

R: 1-amidino-2-ethoxy-3-aminopiperidine (Aeruginosin A), unknown (new Aeruginosin)

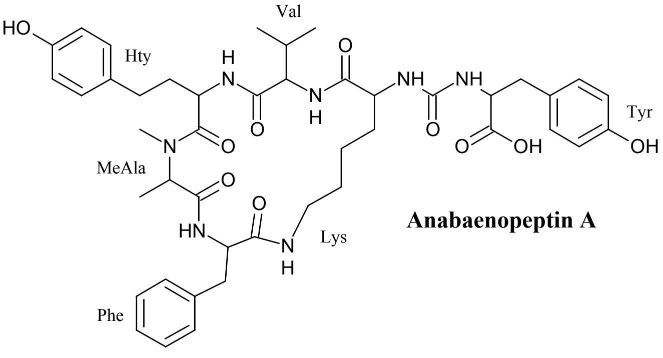

Anabaenopeptin A

Side chain: Tyr in Anabaenopeptin A and Oscillamid Y, Arg in Anabaenopeptin B and F

Val replaced by Ile in Anabaenopeptin F and Oscillamid Y

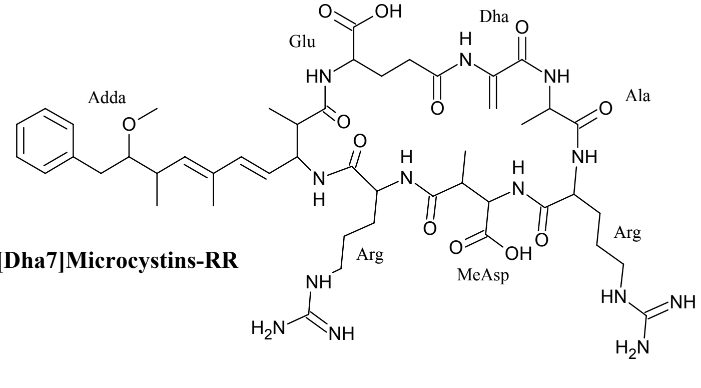

[Dha7]Microcystins-RR

Arg replaced by Leu in [Dha 7]Microcystin-LR

Oscillapeptin G

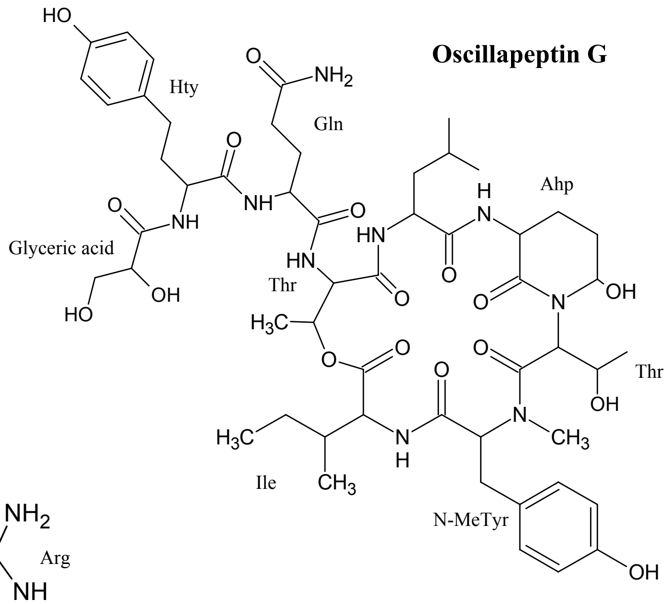

Oscillatorin

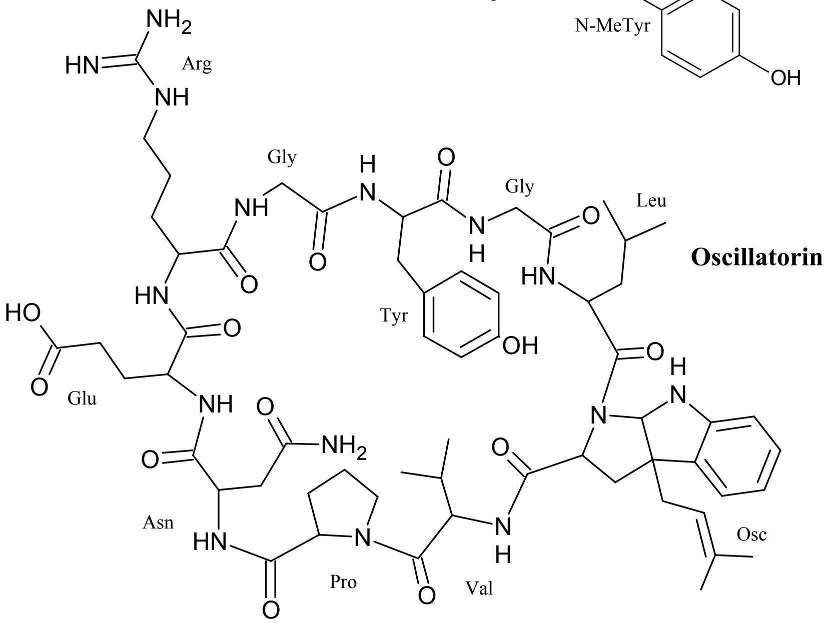

Supplement: Additional file 2 — Oligopeptide structures. Figure S1: Oligopeptide structures of Oscillaginins, Aeruginosins, Anabaenopeptin A, Microcystins-RR, Oscillapeptin G and Oscillatorin, The structure of the putative microviridin is not given since no structural elucidation has been conducted. [file 1471-2164-10-396-S2.pdf]
