# Supplementary material for: A genome-wide analysis of nonribosomal peptide synthetase gene clusters and their peptides in a Planktothrix rubescens strain
Source: BMC Genomics. 2009 Aug 25;10:396. doi: 10.1186/1471-2164-10-396 (PMC2739229; doi:10.1186/1471-2164-10-396)
Supplement: Additional file 3 — NRPS protein sequence data, primers and accession numbers. Table S1: Proteins encoded by the NRPS gene clusters. Table S2: PCR and sequencing primers. Table S3: Accession numbers for the sequences used in the phylogenetic analyses. Figure S1: Variation in the microginin gene cluster. Figure S2: A-domain tree – confirms amino acid activation. Figure S3: Bayesian C-domain tree. Figure S4: E-domains phylogeny. Figure S5: M-domains phylogeny. [file 1471-2164-10-396-S3.pdf]

Additional file 3: NRPS protein sequence data, primers and accession numbers

Table S1: Proteins encoded by the NRPS gene clusters

Protein	AA	Domains/Proteins	Deduced/*In silico* predicted* /function	Sequence similarity/ % Identity	contig
McyD	3910	KS1 AT1 DH1 KR1 ACP1 KS2 AT2 DH2 KR ACP	synthesis of Adda	McyD *Planktothrix* NIVA-CYA 126/8/ 99%	145
McyE	3488	KS AT ACP AMP C1 A1 T1 C2	D-Glu	McyE *Planktothrix* NIVA-CYA 126/8/ 99%	145
McyG	2642	A2 T2 KS4 AT4 KR3 ACP 4	synthesis of Adda	McyG *Planktothrix* NIVA-CYA 126/8/ 99%	12
McyH	585	ABC transporter	ABC transporter	McyH *Planktothrix* NIVA-CYA 126/8/ 99%	12
McyA	1877	A3 T3 C3 A4 T4 E1	Thr and D-Ala	McyA without NMT P. rub 108 CAG38901 /100%	12
McyB	2145	C4 A5 T5 C5 A6 T6	Leu/Arg and Asp-ME	McyB *Planktothrix* NIVA-CYA 126/8/ 98%	12
McyC	1299	C6 A7 T7 TE	Arg	McyC *Planktothrix* NIVA-CYA 126/8/ 99%	12
OciD	687	ABC transporter	ABC transporter	OciD *Planktothrix* NIVA-CYA 116/ 97%	13459
OciA	3944	G T1 C1 A1 T2 C2 A2 T3 C3 A3 T4	Hty , Gln and Thr	peptide synthetase *Anabaena circinalis* 90/66%	13459, 13690, 13586
OciB	4709	C4 A4 T5 C5 A5 T6 C6 A6 T7 C7 A7 M1 T8	Hty, Ahp, Thr, Tyr-ME	OciB *Planktothrix* NIVA-CYA 116/ 91%	13630
OciC	1420	C8 A8 T9 TE	Ile	OciC *Planktothrix* NIVA-CYA 116/ 95%	13630
OciH	134		?	Conseved domains: Ketosteroid isomerase-related protein E=7e-08 polyketide cyclase E=5e-07	13630
OciG	60	Start of halogenase	?	AAZ03553.1 McnD *Microcystis* sp. NIVA-CYA 172/5/56%	13630
AerA	1414	A1 KR T1	Plac moiety	AerA *Planktothrix* NIVA-CYA 126/8/ 94%	27
AerB	1586	Start of halogenase C1 A2 T2 E1	D-Phe	AerB *Planktothrix* NIVA-CYA 126/8/ 81%	27
ORF1	288	Hypotetical protein		ORF3 Aer *Planktothrix* NIVA-CYA 126/8/ 97%	27
AerC	736	Putative oxygenase		AerC *Planktothrix* NIVA-CYA 126/8/ 94%	27
AerD	202	Putative decarboxylase		AerD *Planktothrix* NIVA-CYA 126/8/ 98%	27
AerE	213	Hypotetical protein		AerE Aer *Planktothrix* NIVA-CYA 126/8/ 96%	27
AerF	264	Putative reductase		AerF *Planktothrix* NIVA-CYA 126/8/97% and	27
AerG	1622	T3 A3 T4 C2 T5	Choi moiety	AerG *Planktothrix*	27

				NIVA-CYA 126/8/96%	
AerH	322		Putative dioxygenase	AerH *Planktothrix* NIVA-CYA 126/8/97%	27
AerI	153		putative glycosyltransferase	AerI *Planktothrix* NIVA-CYA 126/8/99%	27
AerK	183		putative glycosyltransferase	AerI *Planktothrix* NIVA-CYA 126/8/97%	27
ORF2	375		Hyp. protein	ORF7 Aer *Planktothrix* NIVA-CYA 126/8/97%	27
AerL	386		Hyp. protein	ORF8 Aer *Planktothrix* NIVA-CYA 126/8/98%	27
AerJ	664		ABC transporter	COG4178: ABC-type transporter, *Nostoc punctiforme* PCC 73102	27
AnaA	2167	A1 T1 C1 A2 T2 E1	Lys and D-Aa ?	ZP_00110699 NRPS *Nostoc punctiforme* PCC 73102/56%	13459
AnaB	1079	C2 A3 T3	Val	ZP_00110698 NPRS *Nostoc punctiforme* PCC 73102/ 65%	13459
AnaC	2576	C3 A4 T4 C4 A5 M1 T5	Hty and Ala-ME	ZP_00110697 NPRS *Nostoc punctiforme* PCC 73102/ 65%	13459
AnaD	1404	C5 A6 T6 TE	Phe	ZP_00110696 NRPS *Nostoc punctiforme* PCC 73102/61%	13459
AnaE	679	ABC transporter	ABC transporter	ZP_00110694 ABC transporter *Nostoc punctiforme* PCC 73102/60%	13459
MicA	3141	KS1 AT1 ACP1 Glutamate-1-semialdehyde aminotransferase-like domain C1 A1 T1	Ahda and Ser	AAY42396.1 HctD *Lyngbya majuscule*/ 58%	13459
MicC	1571	C2 A2 M1 T2	Val-ME	CAC01604 ApdB NRPS cyanopeptolin synthetase *Anabaena* sp.90/59%	13459
MicD	1393	C3 A3 T3 TE	Htyr	CAC01606.1 ApdD NRPS cyanopeptolin synthetase/51%	13459
MicE	668	ABC transporter	ABC transporter	ZP_00110694 *Nostoc punctiforme* ATCC 29133	13459
ORF1	3020	C1 A1 M1 T1 C2 A2 M2 T2	Unknown aa-ME and Unknown aa-ME and	AY212249.1 *mcy Anabaena* sp 90/40%	115, 13820
ORF2	2516	KS1 AT1 KR1 ACP1 TE S	unknown	JamP Lyngbya majuscule/52%	115, 153
ORF3	381	probable butanol dehydrogenase	unknown	NP_488606.1 butanol dehydrogenase *Nostoc* sp. PCC 7120/36%	115, 153
ORF4	299	Hypotetical protein	unknown	YP 320281.1 Phytanoyl-CoA dioxygenase *Anabaena variabilis* ATCC	115, 153

				29413/25%	
ORF5	288	Hypotetical protein	unknown	YP 325491.1 Asp/Glu racemase *Anabaena variabilis* ATCC 29413/25%	115, 153
ORF1	1455	A1 T1 C1	Asp ?	AAZ03552.1 McnC *Microcystis* sp. NIVA-CYA 172/5/32%	13743
ORF2	408	Putative acetate/propionate kinase		ZP_01621769.1 acetate/propionate kinase *Lyngbya* sp. PCC 8106/63%	13743
ORF3	213	Putative phosphoglycerate/bisphosphoglycerate mutase		ZP_00516360.1 Phosphoglycerate/bisphosphoglycerate mutase *Crocosphaera watsonii* WH 8501/64%	13743
ORF4	802	putative phosphoketolase		NP_485524.1 putative phosphoketolase *Nostoc* sp. PCC 7120/79%	13743
ORF5	473	ABC transporter-like		putative exconuclease ATPase subunit *Lyngbya* sp. PCC 8106/ 29%	13743
OscA	619		Hypothetical protein Putative oscillatorin precursor	hypothetical protein L8106_04601 *Lyngbya* sp. PCC 8106/53%	109
	356			thiamine-phosphate pyrophosphorylase *Lyngbya* sp. PCC 8106/73%	109
	256			permease protein of sugar ABC transporter *Lyngbya* sp. PCC 8106/84%	109
	122			hypothetical protein slr5110 *Synechocystis* sp. PCC 6803/32%	109
	203			COG0212: 5-formyltetrahydrofolate cyclo-ligase *Nostoc punctiforme* PCC 73102/60%	109
	299		Peptidase s33 family	2-hydroxy-6-oxohepta-2,4-dienoate hydrolase *Lyngbya* sp. PCC 8106/71%	109
	66			hypothetical protein L8106_30615 *Lyngbya* sp. PCC 8106/64%	109
	431			unnamed protein product *Microcystis aeruginosa* PCC 7806/30%	109

	305			ABC-3 Trichodesmium erythraeum IMS101/82%	109
	612			aspartate kinase *Lyngbya* sp. PCC 8106/76%	109
MdnA	50		Microviridin precursor	Unnamed protein product *Microcystis aeruginosa* PCC7806/59%	13459
MdnC	330		ATP grasp ligase	Hyp. Prot. *Nostoc punctiforme* PCC73102/80%	13459
MdnB	324		ATP grasp ligase	RimK *Nostoc punctiforme* PCC73102/75%	13459
MdnD	179		GCN5-related N-acyltransferase	GCN5-related N-acyltransferase Cyanothece PCC 7822/68%	13459
MdnE	597		ABC transporter	ABC transporter *Microcystis aeruginosa* PCC298/74%	13459
MdnF	283		Methyltransferase	Putative methyltransferase *Microcystis aeruginosa* NIES 843/75%	13459

Table S1: Detailed information of proteins encoded by the NRPS gene clusters including amino acid length (AA), domains and *in silico* predicted function. NRPS and PKS domains are listed with their one letter abbreviations. Ahda = 3-amino-2-hydroxy-decanoic acid and Adda= 3-amino-9-methoxy-10-phenyl-2,6,8-trimethyldeca-4,6-dienoic acid. The most similar sequences identified with BLASTp and % identity to the proteins are listed in addition to native contig.

Table S2: PCR and sequencing primers

Name	Length	Oligo Sequence
98_6R_7R	28	GCACTCTCATCAATAATTCCTGAAAACT
98AGlnR	25	TGCGCTTGATGTTCAGGTAATTTAT
98A2ThrR	26	CATCAGGTTTAATACCCAAAGATTGC
98A1HtyF	28	GATTTACTAAGGACGAGGAGAAAGTTTC
98GlnF2	26	CCAGCTAAATGTGAATTTGTGCAATC
mcyE_F	26	CCATTGACCCAGAGATGACTACAGCT
mcyG_R	28	TCTGAAAATATTCGGTTACATCATTTCC
c8638ociAFalt2	24	GGGATTATTGCTAATCCGAGGGAG
c8636ociAF	27	GGGAGCGAGTTTCTCAATTACCTTTAT
c8636ociA_Ralt2	24	AACCGATGATTGCACTTGAGGATT
c8636_ociA_R	28	GATGATTGCACTTGAGGATTTTGAGTTA
ociAc13630_Ralt2	28	GTTGTGCTTGATGTTGAGGAATAGACTC
ociAc1363_R	26	TGCTTGATGTTGAGGAATAGACTCGA
c100ociA_F	24	TTCTTTAATGCTTATGGGCCAACG

Table S2: Primers used for PCR and sequencing to confirm correct assembly and close gaps between the 454-sequences.

Table S3: Accession numbers for the sequences used in the phylogenetic analyses

**ABC transporter tree (Figure 1)**	**Accession number**
McyH NIVA CYA 98	AM990462
McyH NIVA-CYA 126/8	AJ441056
McyH *Microcystis aerugiosa*	AB032549
NdaI *Nodularia*	AY210783
NcpC *Nostoc*	AY167420
NosG *Nostoc*	AF204805
Uncharachterized ABC transporter *Anabaena variabilis*	NC010852
MicE NIVA CYA98	AM990464
OciD NIVA CYA116	DQ837301
OciD NIVA CYA 205	EU109504
OciD NIVA CYA 98	AM990463
Microcystis McnF NIVA CYA172/5	DQ075244
AerJ NIVA CYA 126	AM071396
AerJ NIVA CYA 98	AM990465
AnaE NIVA CYA 98	AM990463
**A-domain tree (Figure 2 additional file 2)**	**Accession number**
A sp90 McyC A R	AJ536156
A sp90 McyB A RL	AJ536156
P NIVA CYA 126/8 McyB A5 LR	AJ441056
P NIVA CYA 98 McyB A5 LR	AM990462
P NIVA CYA 98 McyC A7 R	AM990462
M PCC7806 McyC A R	AF183408
P NIVA CYA 126/8 McyA A S	AJ441056
M PCC7806 McyA A S	AF183408
A sp90 McyA A S	AJ536156
Psyr syr A1 S	NC007005
N NcpB2 S	AF204805
N NosA2 S	AY167420
N NcpA3 Q	AF204805
A sp90 ApdA1 Q	AJ269505
M NIVA CYA172/5 McnA Q	DQ075244
P NIVA CYA 98 OciA A2 Q	AM990463
P NIVA CYA 126/8 McyA Ad	AJ441056
P NIVA CYA 98 McyA A4 Ad	AM990462
A sp90 McyA A Ad	AJ536156
M PCC7806 McyA Ad	AF183408
P NIVA CYA 98 AnaC A5 A	AM990463
N NosC2 G	AF204805
P NIVA CYA 98 NRPS-like A1 G?	AM990466
N NcpA2 G	AY167420
P NIVA CYA 126/8 McyG F	AJ441056
P NIVA CYA 98 McyG A2 F	AM990462
M PCC7806 McyG F	AF183408
A sp90 McyG F	AJ536156
P NIVA CYA126/8 AerA phenylpyruvate	AM071396
P NIVA CYA 98 AerA A1	AM990465

P NIVA CYA 126/8 McyE A Ed	AJ441056
P NIVA CYA 98 McyE A1 Ed	AM990462
M PCC7806 McyE A Ed	AF183408
A sp90 McyE A Ed	AJ536156
P NIVA CYA 126/8 McyB A6 Dm	AJ441056
P NIVA CYA 98 McyB A6 Dm	AM990462
A sp90 McyB A Dm	AJ536156
M PCC7806 McyB A Dm	AF183408
P NIVA CYA 98 NRPS-like2 A1 asp	AM990467
M NIVA CYA172/5 McnC1 I	DQ075244
P NIVA CYA 98 NRPS-like1 A2 uk	AM990466
P NIES 205 OciB A5 I	EU109504
P NIVA CYA 98 OciB A5 I	AM990463
M NIVACYA172/5 McnE Q	DQ075244
P NIVA CYA 116 OciC A7 I	DQ837301
P NIES 205 OciC A7 I	EU109504
P NIVA CYA 98 OciC A7 I	AM990463
A sp90 ApdD I	AJ269505
N NosA1 I	AF204805
Psyr SupA A3 V	NC007005
P NIVA CYA 98 MicC A2 V	AM990464
P NIVA CYA 98 AnaB A3 V	AM990463
P NIES 205 OciF A2 T	EU109504
P NIVA CYA 116 OciA A2 T	DQ837301
P NIVA CYA98 OciA A3 T	AM990463
P NIVA CYA 98 OciB A5 T	AM990463
P NIVA CYA 98 McyA T	AM990462
M NIVA CYA 172/5 McnB T	DQ075244
A sp90 ApdA2 T	AJ269505
A sp90 ApdB3 T	AJ269505
Psyr SypA A1 T	NC007005
P NIES 205 OciB A6 F	EU109504
P NIVA CYA 116 ociB A6 F	DQ837301
P NIVA CYA 98 ociB A6 Y	AM990463
M NIVA CYA 172/5 McnC4 Y	DQ075244
P NIVA CYA 98 AnaD A6 F	AM990463
A sp90 ApdB4 Y	AJ269505
M NIVA CYA 172/5 McnC3 F	DQ075244
N NcpA1 Y	AY167420
N NosD1 Y	AF204805
N NcpB4 F	AY167420
P NIVA CYA 98 AerB A2 F	AM990465
P NIVA CYA 98 MicD A3 Hty	AM990464
P NIVA CYA 98 AnaC A4 Hty	AM990463
A sp90 ApdB1 Hty	AJ269505
P NIVA CYA 116 OciA A1 Hty	DQ837301
P NIVA CYA 98 OciA A1 Hty	AM990463
P NIES 205 OciA A1 Hty	EU109504

P NIES 205 OciB A4 Ahp	EU109504
P NIVA CYA 116 OciB A4 Ahp	DQ837301
P NIVA CYA 98 OciB A4 Ahp	AM990463
M NIVA CYA172/5 McnC2 Ahp	DQ075244
A sp90 ApdB2 Ahp	AJ269505
P NIVA CYA 98 AnaA A2 uk	AM990463
P NIVA CYA 98 AnaA A1 uk	AM990463
P NIES 205 ociB A3 Hty	EU109504
P NIVA CYA 116 OciB A3 Hty	DQ837301
P NIVA CYA 98 OciB A3 Hty	AM990463
N NosC1 L	AF204805
N NosA4 L	AF204805
M PCC7806 McyB A L	AF183408
P NIVACYA126/8 AerB A2 L	AM071396
N NcpB3 mP	AY167420
N NosA3 mP	AF204805
N NosD2 P	AF204805
P NIVACYA126/8AerG A3 Choi	AM071396
P NIVA CYA 98 AerG A3 Choi	AM990465
Cylindrospermopsis raciborskii AoaB A1 G	EU076463
**C-domain tree (Figure 3 additional file 2)**	**Accession number**
P NIES 205 OciF C2	EU109504
M NIVACYA172/5 McnB C2	DQ075244
N NcpB C4	AY167420
P NIVACYA98 AerG C2	AM990465
P NIVA CYA 98 AnaB C2	AM990463
P NIVA CYA 98 mcyB C4	AM990462
P NIVA CYA 126/8 McyB C	AJ441056
A sp90 McyB C	AJ536156
M PCC7806 McyB C	AF183408
P NIVA CYA 98 McyA C3	AM990462
P NIVA CYA 126/8 McyA C	AJ441056
A sp90 Mcy A C	AJ536156
M PCC7806 McyA C	AF183408
P NIVA CYA 98 McyE C1	AM990462
P NIVA CYA 126/8 McyE C	AJ441056
M PCC7806 McyE C	AF183408
A sp90 McyE C	AJ536156
P NIVA CYA 98 MicA	AM990464
P NIVA CYA 98 McyB C5	AM990462
P NIVA CYA 126/8 McyB C5	AJ441056
A sp90 McyB C2	AJ536156
M PCC7806 McyB C2	AF183408
P NIVA CYA 98 McyE C2	AM990462
P NIVA CYA 126/8 McyE C2	AJ441056
A sp90 McyE C2	AJ536156
M PCC7806 McyE C2	AF183408
P NIVA CYA 98 McyC C6	AM990462

P NIVA CYA 126/8 McyC C	AJ441056
A sp90 McyC C	AJ536156
M PCC7806 McyC C	AF183408
N NosA1C	AF204805
M NIVACYA172/5 McnA C1	DQ075244
N NcpA1C	AY167420
P NIVA CYA 98 NRPS-like2 C1	AM990467
P NIVA CYA 98 AnaC C3	AM990463
P NIVA CYA 98 OciC C7	AM990463
P NIVACYA 116 OciC C7	DQ837301
P NIES 205 OciC C7	EU109504
M NIVACYA172/5 McnE	DQ075244
A sp90 ApdD C	AJ269505
P NIVA CYA 98 MicD C3	AM990464
P NIES 205 OciB C6	EU109504
M NIVACYA172/5 McnC4	DQ075244
P NIVACYA 116 OciB C6	DQ837301
A sp90 ApdB4 C	AJ269505
P NIVA CYA 98 MicC C2	AM990464
P NIES 205 OciA C1	EU109504
P NIVACYA 116 OciA C1	DQ837301
P NIVA CYA 98 AerB C1	AM990465
N NcpB C2	AY167420
N NcpA C3	AY167420
N NcpB C4	AY167420
N NcpB3C	AY167420
N NcpA2C	AY167420
P NIVA CYA 98 OciA C3	AM990463
P NIVA CYA 98 OciA C2	AM990463
P NIVACYA 116 OciA C2	DQ837301
A sp90 ApdA C	AJ269505
A sp90 ApdB C3	AJ269505
N NosD C2	AF204805
N NosC C3	AF204805
N NosC C2	AF204805
N NosA C3	AF204805
N NosA C4	AF204805
N NosA C2	AF204805
N NosD C1	AF204805
A sp90 ApdB C2	AJ269505
A sp90 ApdC C1	AJ269505
P NIES 205 OciB C5	EU109504
P NIVACYA 116 OciB C5	DQ837301
P NIVA CYA 98 OciB C5	AM990463
McnC3	DQ075244
P NIES 205 OciB C4	EU109504
P NIVACYA 116 OciB C4	DQ837301
P NIVA CYA 98 OciB C4	AM990463

McnC2	DQ075244
P NIVA CYA 98 OciA C1	AM990463
P NIVA CYA 98 OciB c3	AM990463
P NIES 205 OciB C3	EU109504
P NIVACYA 116 OciB C3	DQ837301
M NIVACYA172/5 McnC1	DQ075244
P NIVA CYA 98 NRPS-like1 C2	AF204805
N NosC1	AF204805
P NIVA CYA 98 AnaA c1	AM990463
P NIVA CYA 98 AnaD C5	AM990463
P NIVA CYA 98 AnaC C4	AM990463
P NIVA CYA 98 NRPS-like1 C1	AM990466
P NIVA CYA 98 AerG C3	AM990465
**E-domain tree (Figure 4 additional file 2)**	**Accession number**
McyA E1 NIVA CYA 98	AM990462
McyA E1 *Microcystis aeruginosa*	AB032549
AerB E1 NIVA CYA 98	AM990465
NcpA E1 Nostoc	AY167420
OciA E NIES 205	EU109504
AnaA e1 NIVA CYA 98	AM990463
MycB E2 Bacillus subtilis	AF184956
MyB E1 Bacillus subtilis	AF184956
MyC E Bacillus subtilis	AF184956
GrsA E Bacillus brevis	M29703
TycA E1 Bacillus brevis	AF004835
TycB E1 Bacillus brevis	AF004835
LicA E Bacillus licheniformis	U95370
**M-domain tree (Figure 4 additional file 2)**	**Accession number**
OciB M1 NIES 205	EU109504
ociB M1 NIVA CYA 116	DQ837301
OciB M1 NIVA CYA 98	AM990463
MicC M1 NIVA CYA 98	AM990464
AnaC M1 NIVA CYA 98	AM990463
contig115 ORF2 M2 NIVA CYA 98	AM990466
contig115 ORF1 M1 NIVA CYA 98	AM990466

Table S3: Accession numbers for the sequences used in ABC transporter (Figure 1), A-domain (Figure 2 additional file 2), C-domain (Figure 3 additional file 2), E-domain (Figure 4 additional file 2) and M-domain (Figure 4 additional file 2) phylogenies. The sequences are listed in the order they appear in the tree (top to bottom).

Figures:

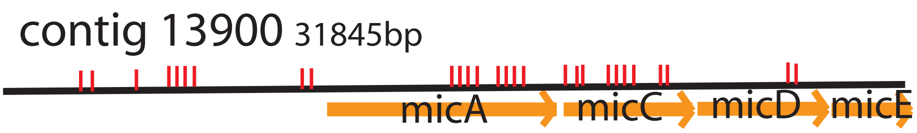

Figure S1: **Variation in the microginin gene cluster.** The contig (#13900) containing the microginin gene cluster. The contig display high sequence depth (45x) and contains 28 variable sites. The sites indicated in red illustrate polymorphic positions where more than two reads display the same nucleotide variant.

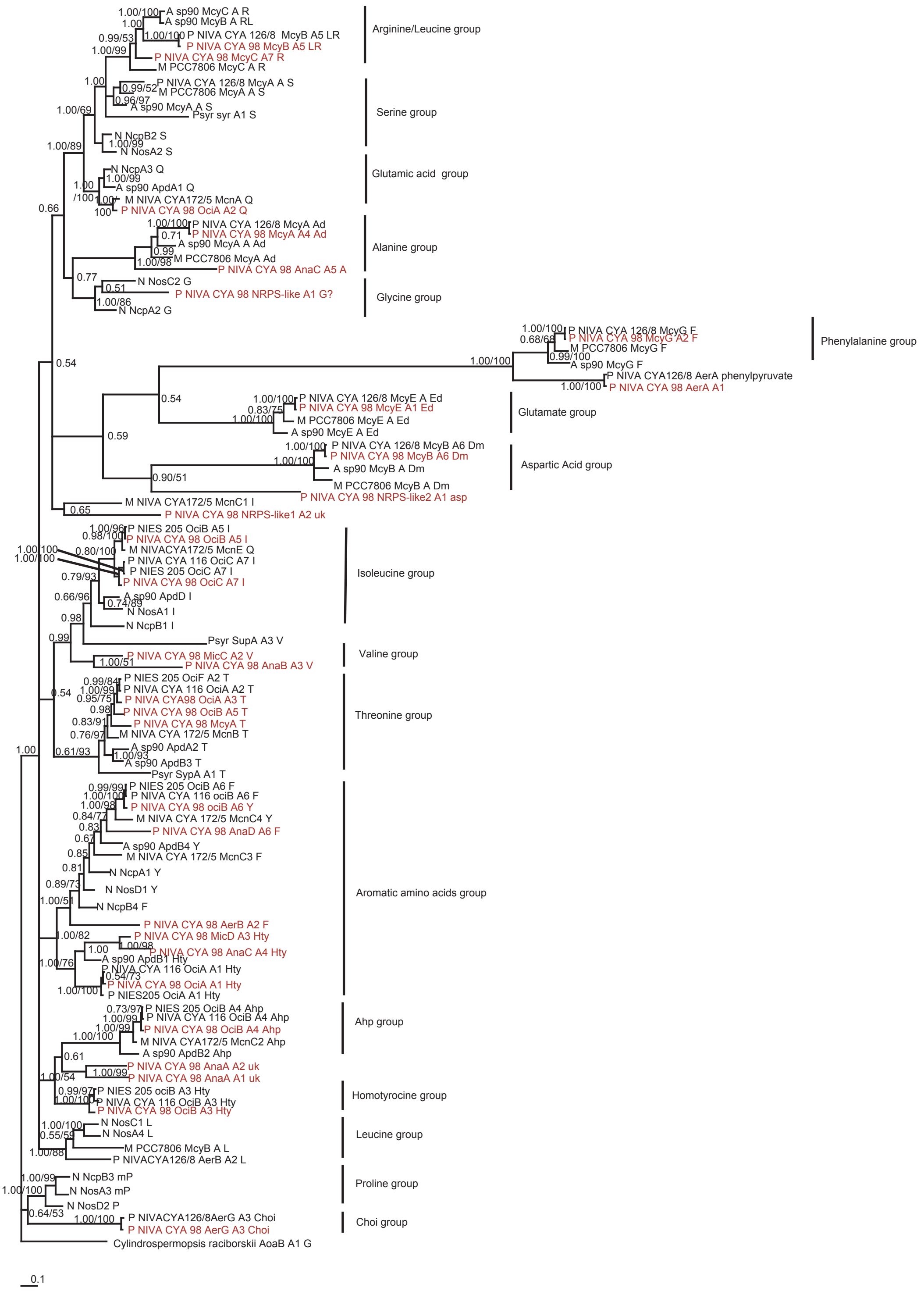

Figure S2: **A-domain tree – confirms amino acid activation**. Optimal protein evolution model (RtRev) and gamma-shaped distribution were utilized in bayesian inference analysis. The MCMC chains were carried out for 4 million generations with sampling of trees every 100-generation, removing the first 3000 trees. Neighbor-Joining (NJ) trees were constructed using MEGA 3.1 with default settings (Poisson correction as the amino acid substitution model) (41). Support values for the nodes are Bayesian posterior probability and NJ bootstrap replicates above 50%. Genus origin is denoted with first letter abbreviations (P=*Planktothrix*, M=*Microcystis*, A=*Anabaena* and N=*Nostoc*). The A domains are numbered in according to the position in the gene cluster. The putative activated amino acid shown in one-letter abbreviations (Ad =D-alanine, Hty=homotyrosine, Ed=D- glutamate and Dm=methylaspartic acid). The accession number for each sequence is listed in Table 3 Additional file 2.

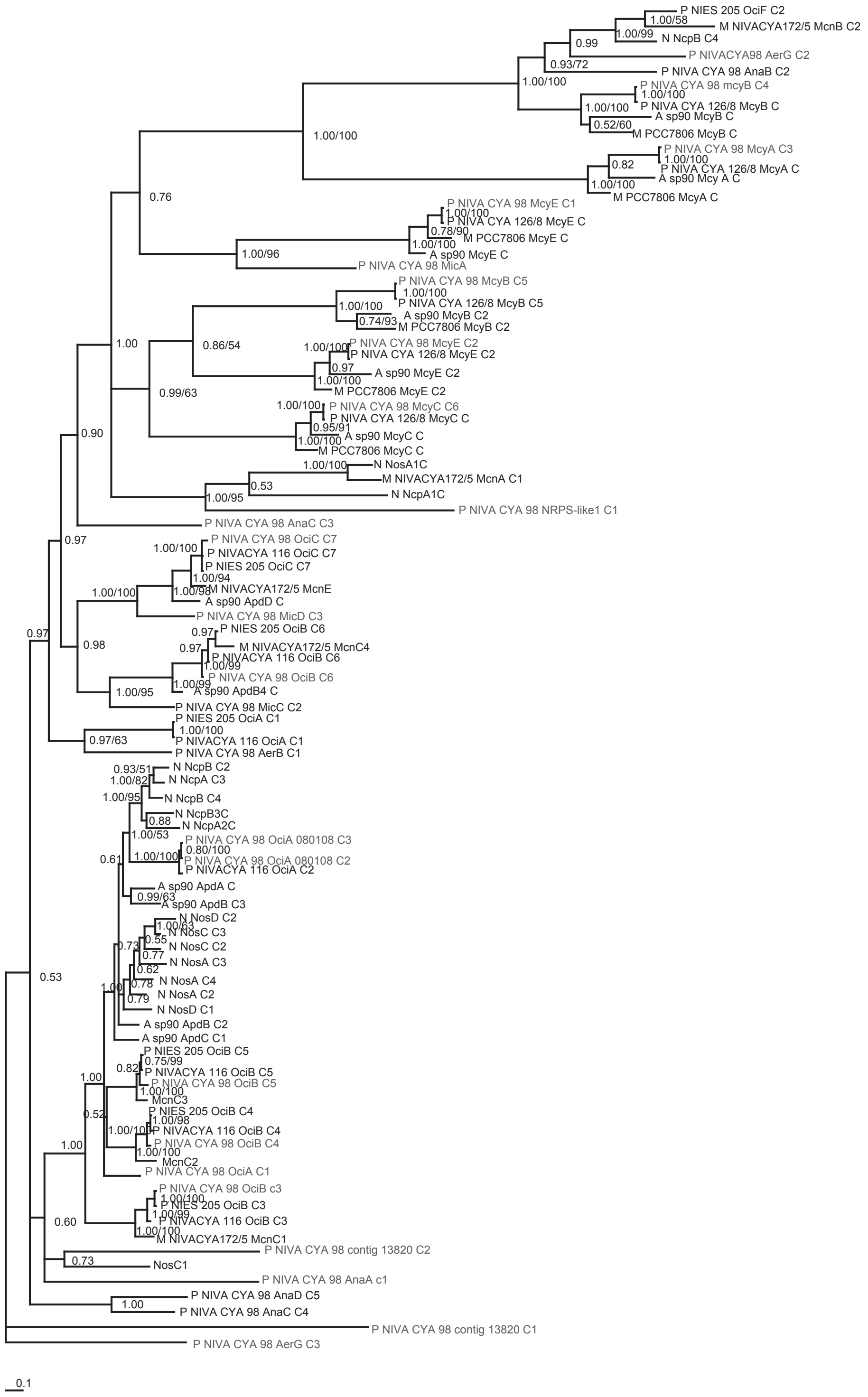

Figure S3: **Bayesian C-domain tree**. The C-domain phylogeny shows clustering according to gene cluster function/position and indicates E-domains downstream of D-amino acid C-domains. The analysis of the C-domains was performed as described for the A-domains with Mtmam as optimal evolution. Support values for the nodes are Bayesian posterior probability and NJ bootstrap replicates above 50%. Genus origin is shown with first letter abbreviations (P = *Planktothrix*, M = *Microcystis*, A = *Anabaena* and N = *Nostoc*), and the C-domains are labelled in numerical order according to direction of transcription. The accession number for each sequence is listed in Table 3 Additional file 2.

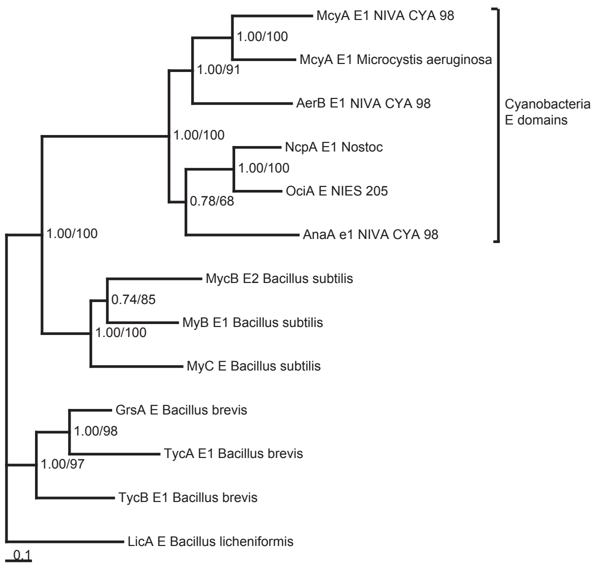

Figure S4: **E-domains phylogeny**. The tree shows clustering according to gene clusters. Bayesian analyses were performed as described for the A-domains with WAG as optimal evolution model. Support values for the nodes are Bayesian posterior probability and NJ bootstrap replicates above 50%. E-domains without strain names originates from *Planktotrix* CYA 98. The accession number for each sequence is listed in Table 3 Additional file 2.

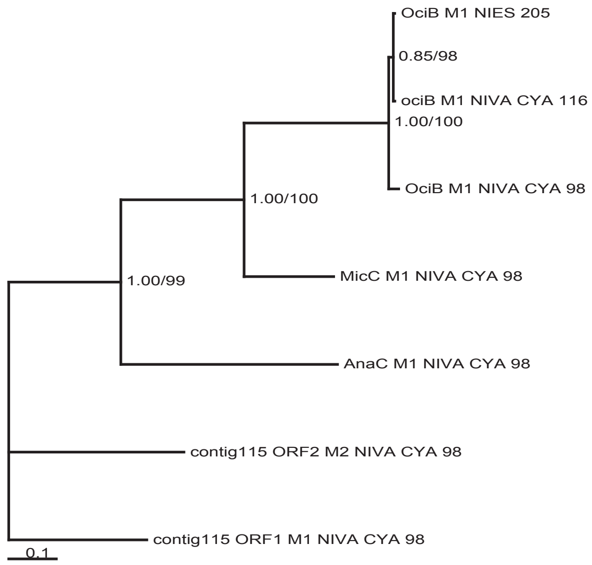

Figure S5: ***Planktothrix* CYA98 M-domains phylogeny.** The tree shows clustering according to gene clusters. Bayesian analyses were performed as described for the A-domains with TrRev as optimal evolution model. Support values for the nodes are Bayesian posterior probability and NJ bootstrap replicates above 50%. The accession number for each sequence is listed in Table 3 Additional file 2.
