# Supplementary material for: A genome-wide analysis of nonribosomal peptide synthetase gene clusters and their peptides in a Planktothrix rubescens strain
Source: BMC Genomics. 2009 Aug 25;10:396. doi: 10.1186/1471-2164-10-396 (PMC2739229; doi:10.1186/1471-2164-10-396)
Supplement: Additional file 4 — Splits decomposition analyses. Figure S1: a) Splits decomposition analysis of A-domains from Planktothrix CYA 98. b) Splits decomposition analysis of C-domains from Planktothrix CYA 98. c) Split decomposition analysis of M-domains from Planktothrix CYA 98. d) Splits decomposition analysis of E-domains. e) Splits decomposition analysis of ABC transporters. [file 1471-2164-10-396-S4.pdf]

Additional file 4: Splits decomposition analyses

Figure S1: Splits Trees of NRPS domains from *Planktothrix* CYA 98 were constructed using SplisTree4 with 1000 bootstrap replica. The splits decomposition analyses and Phi test did not find statistically significant evidence for recombination in any of the domains analyzed.

a) Splits decomposition analysis of A-domains from *Planktothrix* CYA 98
b) Splits decomposition analysis of C-domains from *Planktothrix* CYA 98
c) Split decomposition analysis of M-domains from *Planktothrix* CYA 98
d) Splits decomposition analysis of E-domains
e) Splits decomposition analysis of ABC transporters

a) Splits decomposition analysis of A-domains from *Planktothrix* CYA 98

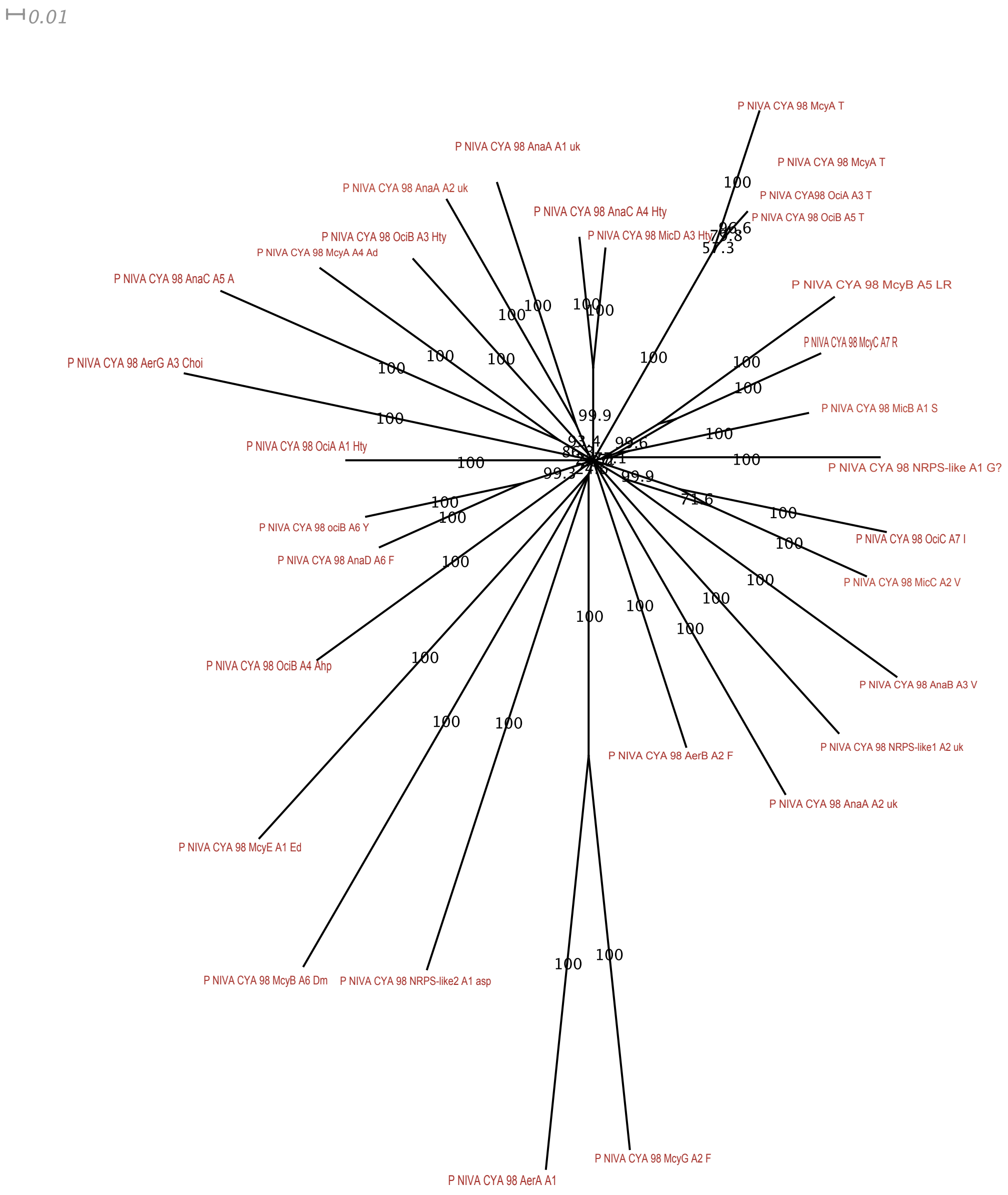

The phi test did not find statistically significant evidence for recombination (p = 0.99)

b) Splits decomposition analysis of C-domains from *Planktothrix* CYA 98

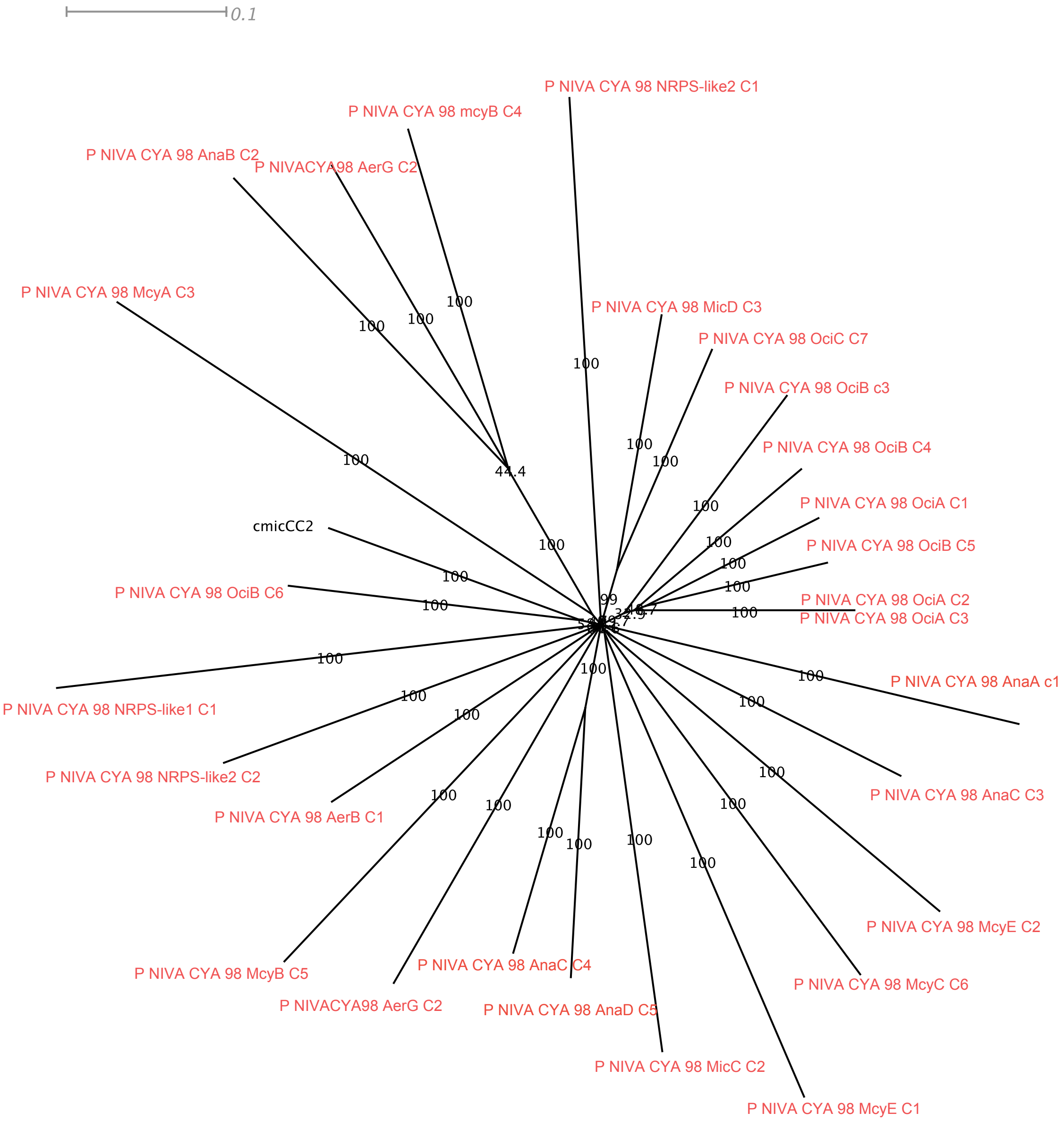

The phi test did not find statistically significant evidence for recombination (p = 0.99)

c) Split decomposition analysis of M-domains from *Planktothrix* CYA 98

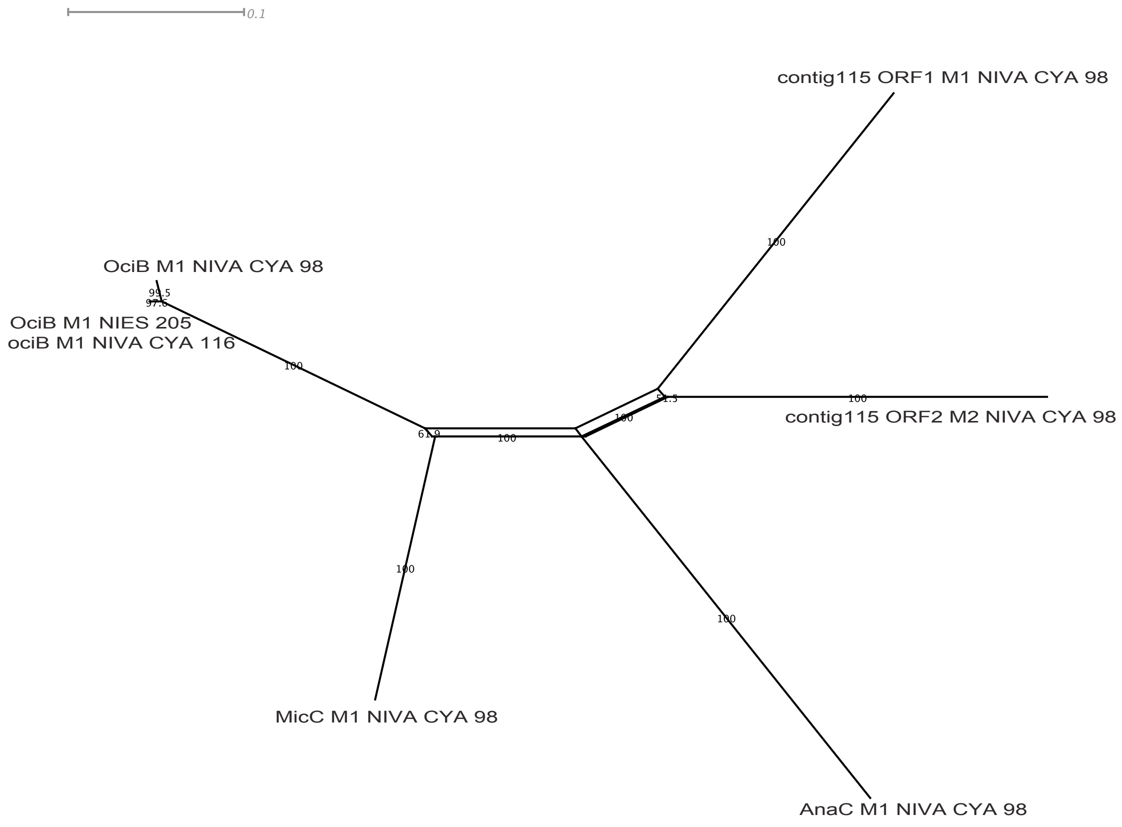

The phi test did not find statistically significant evidence for recombination (p = 0.95)

d) Splits decomposition analysis of E-domains

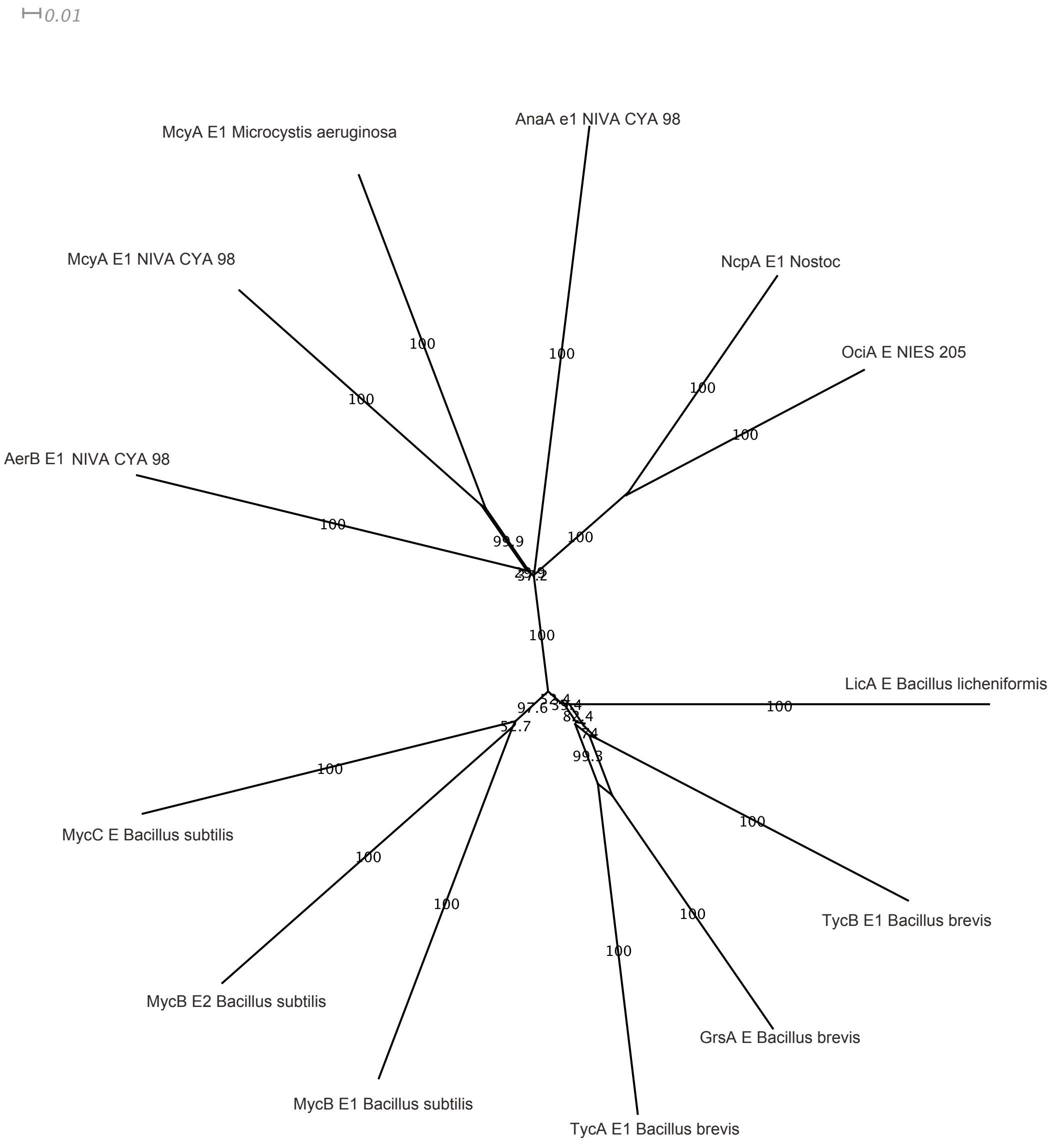

The phi test did not find statistically significant evidence for recombination (p = 0.50)

e) Splits decomposition analysis of ABC transporters

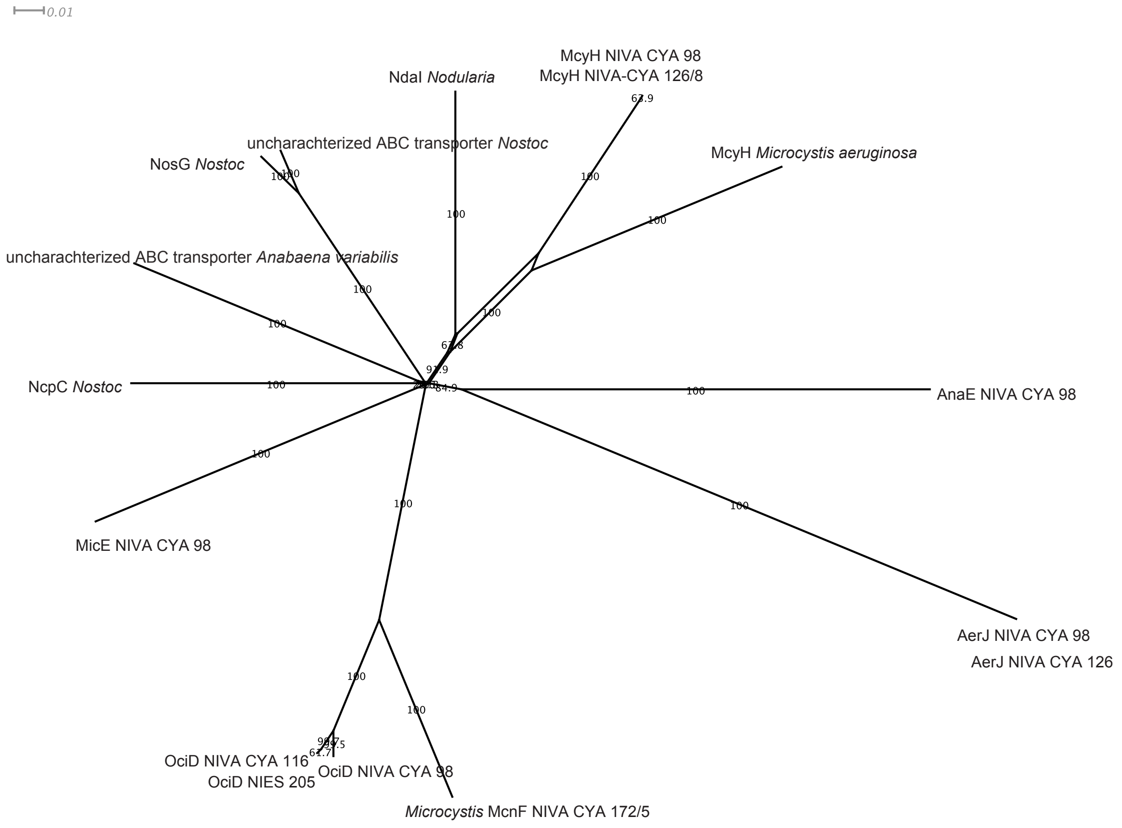

The phi test did not find statistically significant evidence for recombination (p = 0.86)
